# Roles of microRNA-192 in diabetic nephropathy: the clinical applications and mechanisms of action

**DOI:** 10.3389/fendo.2023.1179161

**Published:** 2023-06-15

**Authors:** Xiaoqing Wan, Jian Liao, Hongting Lai, Shilong Zhang, Jianling Cui, Chunyan Chen

**Affiliations:** ^1^ Department of Nephrology, Taizhou Central Hospital (Taizhou University Hospital), Taizhou, Zhejiang, China; ^2^ Department of Nephrology, Jiaxing Hospital of Traditional Chinese Medicine, Jiaxing, Zhejiang, China; ^3^ Clinical Medical College, Tianjin Medical University, Tianjin, China; ^4^ Clinical Medical College, Zhejiang Chinese Medical University, Hangzhou, Zhejiang, China

**Keywords:** MicroRNA-192, diabetic nephropahy, biomarker, Zeb1, mechanism

## Abstract

Diabetic nephropathy (DN) is one of the most common and intractable microvascular complications of diabetes worldwide, serving as the main cause of terminal renal disease. Due to the lack of early specific symptoms and diagnostic markers, DN severely threatens the sufferer’s life. MicroRNA-192 (miR-192) was early identified in human renal cortical tissue and stored and excreted in urine as microvesicles. MiR-192 was found to be involved in the development of DN. For the first time, the present review summarized all the current evidence on the topic of the roles of miR-192 in DN. Finally, 28 studies (ten clinical trials and eighteen experimental studies) were eligible for thorough reviewing. Most of the clinical trials (7/10, 70%) indicated miR-192 might be a protective factor for DN development and progression, while the majority of experimental studies (14/18, 78%) suggested miR-192 might be a pathogenic factor for DN. Mechanistically, miR-192 interacts with various direct targeted proteins (i.e., ZEB1, ZEB2, SIP1, GLP1R, and Egr1) and signaling cascades (i.e., SMAD/TGF-β and PTEN/PI3K/AKT), together contribute to the pathogenesis of DN through epithelial-to-mesenchymal transition (EMT), extracellular matrix deposition, and fibrosis formation. The current review highlights the dual role of miR-192 in the development of DN. Low serum miR-192 expression could be applied for the early prediction of DN (the early stage of DN), while the high miR-192 level in renal tissues and urine may imply the progression of DN (the late stage of DN). Further investigations are still warranted to illustrate this inconsistent phenomenon, which may facilitate promoting the therapeutic applications of miR-192 in predicting and treating DN.

## Introduction

Diabetic nephropathy (DN), a serious renal disease, is found to be correlated with the development of proteinuria, glomerular enlargement, reduction in glomerular filtration, and renal fibrosis (1). It affects roughly 9% of the global adult population in those suffering from either type 1 (T1DM) or type 2 diabetes mellitus (T2DM). In China, the prevalence of diabetes in adults was recorded at about 11% (2), while the prevalence of DN was up to 21.8% (3). There is a positive association between DN and the risk of the development of terminal renal disease (4). In diabetic patients with terminal kidney disease, the five-year survival rate is only 20%. There is a significant increase in deaths and morbidity associated with DN-associated heart failure with unchanged ejection fraction (5, 6). Renal replacement therapy is necessary in a substantial proportion of cases due to there are few effective treatments. At present, the therapeutic strategies for DN aim to reduce its progression but most of them turned out to be ineffective (7). As a result, an effective, rapid, and non-invasive method for detecting DN early and predicting its prognosis is critical. In the past two decades, multiple microRNAs (miRNAs) have been identified to involve in the pathophysiological action in DN.

MiRNAs are short, endogenous, and noncoding RNA molecules consisting of 19-24 nucleotides. A miRNA achieves its biological function by binding to the 3’ untranslated region of a target gene mRNA. By promoting the degradation of mRNA or causing translational repression, miRNAs effectively regulate gene expression post-transcriptionally (8, 9). MiRNAs are involved in the regulation of multiple cellular biological processes, either physiologic or pathologic conditions (e.g., proliferation, differentiation, programmed death, apoptosis, or passive cell death) (8). The dysregulation and aberrant expression of several miRNAs are also found to be associated with the development and progression of DN, such as miR-21, miR-126, miR-29, miR-192, miR-214, miR-342, and miR-192 (10, 11). According to the current literature, miR-192 is one of the most investigated miRNAs with aberrant expression in DN. Mounting clinical studies demonstrated that abnormal miR-192 expression level was detected in the majority of patients with DN. In addition, the molecular mechanisms of miR-192 action are found to be complex and multidirectional in the development of DN.

Since the pivotal role of miR-192 in DN has received increasing attention from the investigators, it is, therefore, necessary to summarize all the current evidence on this topic through a comprehensive review, which may help to develop a better understanding of the prognostic and predictive role of miR-192 in DN.

## Overview of miR-192

MiRNAs are small noncoding RNAs that inhibit messenger RNAs by binding to their 3’-UTRs (12). MiR-192 is a conserved miRNA that is profoundly expressed in various mammalian cell types. Human miR-192 derives from a coding gene located on chromosome 11. It produces two mature transcripts, including miR-192 (miR-192-5p) and miR-192* (miR-192-3p) (13). miR-192 is found to involve in the regulation of different physiological and pathological processes, including epigenetics, differentiation, proliferation, apoptosis, epithelial-mesenchymal transition (EMT), angiogenesis, metabolism, inflammatory responses, oxidative stress, and drug resistance (14, 15). These biological functions are derived from the inhibition of the miR-192-targeted genes, such as mRNA degradation and repression of protein translation. Given its key role in cellular processes, dysregulation of miR-192 is considered to contribute to the genesis of multiple human diseases, including respiratory system diseases (i.e., asthma, nasopharyngeal carcinoma, lung cancer), digestive system diseases (i.e., hepatic disorders, esophageal, colorectal, and gastric cancers), circulatory system diseases (i.e., myocardial fibrosis, myocardial infarction, cardiac injury), urinary system diseases (i.e., bladder cancer, kidney injury), reproductive system diseases (i.e., breast, cervical, ovarian, and prostate cancer), endocrine system (i.e., diabetes, hyperglycemia, and insulin resistance), and nervous system diseases (i.e., Alzheimer’s disease, amyotrophic lateral sclerosis, tuberous sclerosis, and peripheral nerve injury) (14–19). Functionally, miR-192 induces post-transcriptional gene silencing by binding to the 3’-UTR to regulate its targeted genes. miR-192’s conserved enhancer elements contain the different binding sites for multiple targeted genes, i.e., CCNB1, Nidogen-1, RAB1A, and ACVR2B (20–23). According to the software “TargetScan Human” (https://www.targetscan.org/), there are 3,483 transcripts with sites available, indicating more than 3,000 predicted targets have been found. However, we should know that relatively few targeted genes for miR-192 were experimentally validated.

The expression levels of miRNAs can be regulated by other non-coding RNAs, such as long non-coding RNAs (lncRNAs) and circular RNAs (circRNAs). According to the current evidence, both lncRNAs and circRNAs can act as upstream regulators that modulate the expression of miR-192. The discovered lncRNAs interacted with miR-192 include KCNQ1OT1, FTX, WAC-AS1, WAC-AS1, PTTG3P, and STEAP3-AS1 (15, 24–26). The discovered circRNAs include circ_0000189, circKIF5B, circ-SWT1, and circHIPK3 (27–30). These lncRNAs and circRNAs exert their biological functions by sponging miR-192 and regulating miR-192-targeted genes.

Currently, no relevant review article has been published for summarizing the clinical implications and the molecular mechanisms of miR-192 in DN. As a result, we conducted this comprehensive review based on the current evidence.

## Literature search

To identify the relevant studies investigating the role of miR-192 in DN, a literature review was conducted on each of the six commonly used databases, including. MEDLINE (PubMed), EMBASE, Cochrane Library, Google Scholar, Web of Science, and the PsychINFO, The searching strategy in MEDLINE by using the keywords was: ((((((((((((((((((“Diabetic Nephropathies”[Mesh]) OR (Nephropathies, Diabetic)) OR (Nephropathy, Diabetic)) OR (Diabetic Nephropathy)) OR (Diabetic Kidney Disease)) OR (Diabetic Kidney Diseases)) OR (Kidney Disease, Diabetic)) OR (Kidney Diseases, Diabetic)) OR (Diabetic Glomerulosclerosis)) OR (Glomerulosclerosis, Diabetic)) OR (Intracapillary Glomerulosclerosis)) OR (Nodular Glomerulosclerosis)) OR (Glomerulosclerosis, Nodular)) OR (Kimmelstiel-Wilson Syndrome)) OR (Kimmelstiel Wilson Syndrome)) OR (Syndrome, Kimmelstiel-Wilson)) OR (Kimmelstiel-Wilson Disease)) OR (Kimmelstiel Wilson Disease)) AND (((((“MIRN192 microRNA”) OR (miR-192)) OR (microRNA-192)) OR (hsa-mir-192)) OR (miR-192-5p)). Furthermore, a review of the reference list was conducted to identify more relevant studies.

**Figure 1:**
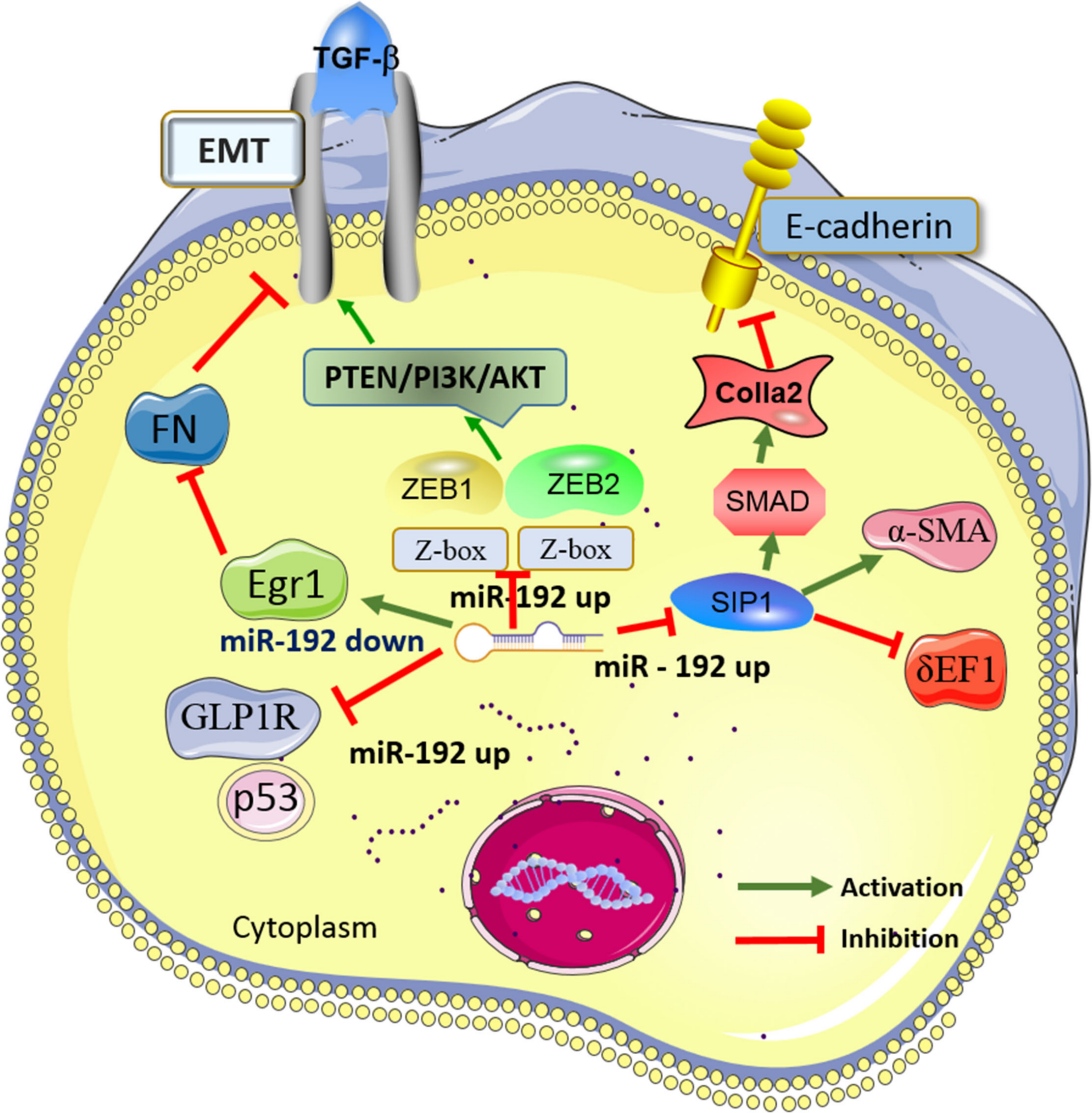
Main mechanisms of miR-192 in diabetic nephropathy (DN). MiR-192 exerts the biological effects in DN by interacting with its target genes (i.e., ZEB1, ZEB2, SIP1, GLP1R, and Egr1) and the signaling cascades (i.e., SMAD/TGF-β and PTEN/PI3K/AKT). DN, Diabetic nephropathy; miR-192, MicroRNA-21; PTEN, phosphatase and tensin homolog deleted in chromosome 10; TGF-β, Transforming growth factor-β; EMT, epithelial-to-mesenchymal transition; GLP-1R, Glucagon-like peptide-1 receptor; ZEB1, Zinc finger E-box-binding homeobox 1; Egr1, Early growth response factor 1; MCP-1,Monocyte chemotactic protein-1; SIP1,Smad-interacting protein 1.

Finally, twenty-eight studies (31–57) (58) published in 2007-2022 were included in this review for further analysis. A routine data collection form was used to collect relevant information from the included studies, e.g., the first author name, article publication year, research subject (patients, cells, or animals), expression of miR-192 (up-regulation or down-regulation), involved molecular mechanisms, target genes, and the main findings within each eligible study. The study design for the 28 included studies was either clinical trials (10 studies) or experimental studies (18 studies). The sample size of the clinical trials ranged from 6 to 602 participants. Diverse clinical specimens were applied in the clinical studies, including serum, urine, and kidney tissues. According to the experimental studies, miR-192 was found to interact with various direct targeted proteins (i.e., ZEB1, ZEB2, SIP1, GLP1R, and Egr1) and signaling cascades (i.e., SMAD/TGF-β and PTEN/PI3K/AKT), together contributed to the pathogenesis of DN through epithelial-to-mesenchymal transition (EMT), extracellular matrix deposition, and fibrosis formation. The characteristics of eligible studies reporting miR-192 in DN were listed in **Tables 1**, **2**. **Figure 1** showed the main molecular mechanisms of miR-192 expression in the development of DN.

**Table 1:** Summary of miR-192 in diabetic nephropathy (DN) reported in clinical trials.

Study/Reference	Research objects	MiR-192 expression	Involved Mechanism	Target Gene	Main findings
Clinical trials (ten studies)					
Chien et al., 2016	Patients (n=50)	No significant different in serum	Clinical study	NA	No significant difference was observed in the miR-192 expression between T2DM subjects with and without DN. However, serological miR-192 differed between microalbuminuria and overt proteinuria groups (P=0.0138), indicating that miR-192 might predict late DN progression.
Jia et al., 2016	Patients (n=602)	Up in urine	Clinical study	NA	The urine extracellular vesicles level of miR-192 was significantly higher in patients with DN, especially in those with albuminuria. Urinary extracellular vesicles miR-192 might serve as a biomarker of the early stage of DN.
Ma et al., 2016	Patients (n=464)	Down in serum	Clinical study	NA	The miR-192 in the albuminuria group was significantly lower than the controls (*P*< 0.05). The expression of miR-192 was negatively correlated with TGF-*β*1, FN, and UACR. There is positive association between miR-192 and nephritic fibrosis in DN.
Yang et al., 2017	Patients (n=283)	Decreased in serum, increased in urine	Clinical study	NA	The miR-192 level in serum decreased, whereas in urine it increased with the progression of DN. Both serum and urinary miR-192 could be a potential biomarker of DN.
KAFAJI et al., 2018	Patients (n=85)	Decreased in serum	Clinical study	NA	Lower miR-192 expression was observed in DN patients compared with healthy controls (*P*< 0.05). Blood-based miR-192 might serve as an effective biomarker for early detection of DN.
Tayel et al., 2019	Patients (n=229)	Down in serum	Clinical study	NA	Significant lower expression levels of both miR-192 and miR-126 was observed in patients with DN than the controls (all P<0.05).
Smith et al., 2021	Patients (n=6)	Down in urine	Clinical study	NA	miR-192 expression fell from a 1.54-fold change in the control cohort in DN patients.
Akpınar et al., 2022	Patients (n=150)	Down in serum	Clinical study	NA	The expression of miR-192-5p was significantly lower in the DN group (P=0.027) than the controls, indicating that miR-192-5p might be associated with the development of DN.
Gong et al., 2022	Pediatric patients (n=79) and HK-2 cells	Up in serum	Increased miR-192 and decreased Alpha-Klotho (KL)	KL	miR-192 expression negatively correlated with KL levels in pediatric patients with prolonged duration of diabetes. Inhibiting miR-192 mitigated oxidative stress, inflammation, and senescence in HK2 cells.
Ren et al., 2022	Patients (n=436)	Down in serum	Downregulated miR-192 and increased TGFβ1	NA	miR-192 interacted with some other miRNAs, their target genes mainly revolve around PTEN, PI3K/Akt, and MAPK signaling pathways.

NA, Not available.

**Table 2:** Summary of miR-192 in diabetic nephropathy (DN) reported in experimental studies.

Study/Reference	Research objects	MiR-192 expression	Distribution of miR-192	Involved Mechanism	Target Gene	Main findings
Kato et al. 2007	Mouse and mesangial cells	Up	Glomeruli	Downregulated SIP1 and δEF1; increased Col1a2; Derepression at E-box elements	SIP1	TGF-β lead to the down-regulation of SIP1 (via miR-192) and δEF1 could cooperate to reinforce Col1a2 expression *via* derepression at E-box elements.
Krupa et al. 2010	Patient tissue and HK-2 cells	Down	Tubules and glomeruli	Upregulated ZEB1 and ZEB2; decreased E-cadherin; Enhancing TGF-β	ZEB1 and ZEB2	Downregulated miR-192 level increased fibrosis and declined GFR in diabetic nephropathy, which might induced by enhancing TGF-β–mediated downregulation of E-cadherin in proximal tubular cells.
Putta et al. 2012	Mouse and cells	Up	Glomeruli	Decreased Zeb1/2 *via* the TGF-β signaling	ZEB1 and ZEB2	Locked nucleic acid (LNA)–modified inhibitor of miR-192 dramatically increased Zeb1/2 expression and reduced the level of collagen, TGF-b, and fibronectin.
Chen et al. 2012	Mouse and mesangial cells	Down	Glomeruli	NA	NA	miR-192 was one of the remarkably decreased microRNAs in diabetic mice.
Mu et al. 2013	Mouse and cells	Up	Glomeruli	NA	NA	miR-192 was significantly upregulated under diabetic conditions but did not involve in the phenotypic transition of DN.
Deshpande et al. 2013	Mouse, cells, and patients	Up	Glomeruli	Increased p53, decreased ZEB2	ZEB2	TGF-b1–induced feedback amplification circuit between p53 and miR-192/ZEB2 contribute to the pathogenesis of DN
Kato et al. 2014	Mouse and cells	Up	Glomeruli	Acetylation of Ets-1 and histone H3; Activation of Akt and p300	NA	Diabetic condition increased miR-192 expression *via* acetylation of Ets-1 and activation of Akt and phosphorylate p300, which in turn acetylates Ets-1 and histone H3 in the glomeruli of diabetic mice.
Oghbaei et al. 2015	Rats	Down	Renal tissues	NA	NA	Exercise significantly upregulated miR-192 expression in the kidney of diabetic rats compared to the healthy group. Exercise could prevent from the expression changes in miR-192 and might be help to prevent the progression of DN.
Liu et al. 2018	Rats and HK-2 cells	Down	Renal tissues	Increased Egr1; Decreased TGF-β1 and FN	Egr1	miR-192 could inhibit the progression of DN and protect DN rats from renal interstitial fibrosis *in vitro* and *in vivo*, which might be mediated by decreasing TGF-β1 and FN.
Jia et al. 2018	HK-2 cells	Up	High glucose-induced renal tubular epithelial cells	Exendin-4 upregulated p53 expression	GLP1R	Exendin-4 downregulated cellular and secreted miR-192, therefore to increase the expression of GLP1R in a p53-dependent manner, which might ameliorate renal fibrosis.
Mao et al. 2019	Rats and RMCs cells	Up	Renal tissues	Astragaloside IV decreased TGF-β1, Smad3, α-SMA and collagen type 1, and elevated Smad7	SIP1	Astragaloside IV downregulates the expression of miR−192 and thus exerted the therapeutic effect on DN, which might be associated with repression of excessive mesangial proliferation and renal fibrosis *via* the TGF-β1/Smad/miR-192 pathway.
Ebadi et al. 2019	Rats	Up	Glomeruli	Spironolactone, captopril, and their combination might decrease the miR-192 expression.	None	Spironolactone, captopril, and their combination could improve DN by targeting miR-192 and miR-29 family.
Yu et al. 2019	Mouse	Up	Glomeruli	Metformin or alcohol extract of Coreopsis tinctoria Nutt (AC) significantly decreased the expression of miR-192	ZEB2	The protective effect of AC on DN might be associated with the reduction of the expression level of miR-192 and upregulation of ZEB2.
Chen et al. 2019	Glomerular mesangial cells	Up	High Glucose-Induced glomerular mesangial cells	Overexpressed miR-192 and reduced Zeb1	Zeb1	Under high glucose, miR-192 was upregulated to elevate the expression of inflammatory factor MCP-1 by suppressing Zeb1 expression.
Mojadami et al. 2021	Mouse	Up	Glomeruli	Gallic acid reduced miR-192 and Nrf2 expression	NA	Gallic acid improved DN induced by methylglyoxal *via* decreasing miR-192-associated with endoplasmic reticulum stress and fibrosis.
Jia et al. 2021	Rats and HK-2 cells	Up	Renal tissues	Increased miR-192-5p and downregulated GLP-1R expression	GLP-1R	Icariin and dihydrotestosterone improved diabetic renal tubulointerstitial fibrosis by restoring autophagy *via* the miR-192-5p/GLP-1R pathway.
Rafiee et al. 2022	Rats and kidney stem cells (KSCs)	Up	Glomeruli	Downregulated miR-192; increased TGF-β and IL-1β expression	NA	KSCs administration decreased the expression of TGF-β, IL-1β, and miR-192, meanwhile suppressed phosphorylation of Smad2 and Smad3 in DN.
Keyhanmanesh et al. 2022	Rats	Up	Renal tissues	Increased TGF-β and miR-192	SIP1	TGF-β and miR-192 expression significantly elevated and SIP1 decreased in the DN group, while administration of troxerutin and insulin reversed this tendency.

SIP1, Smad-interacting protein 1; TGF-β, Transforming growth factor-β; NA, Not available; GLP-1R, Glucagon-like peptide-1 receptor; Zeb1, Zinc finger E-box-binding homeobox 1; Egr1, Early growth response factor 1; MCP-1, Monocyte chemotactic protein-1.

## Clinical significances of miR-192 in DN

Ten included clinical studies provided clinical information on miR-192 expression levels in DN. Most of these clinical trials (eight studies, 7/10, 70%) demonstrated that miR-192 was down-regulated in adult DN patients. Inconsistent with this finding, one included study (39) reported an opposite result that a high level of miR-192 in serum was found in pediatric DN patients as compared to the healthy controls. One study (31) showed that no significant difference was observed in the miR-192 expression between T2DM subjects with and without DN. Two studies reported the expression of miR-192 in urine. One study showed the results in both serum and urine.

### miR-192 expression in the serum of patients with DN

Eight included studies investigated the expression level of miR-192 in venous blood collection (serum) of DN patients. Six out of eight studies (75%) reported the miR-192 expression was down-regulated in patients with DN.

The pathogenesis of DN causes by various factors, such as high glucose and lipid metabolism disorder, which may induce extracellular matrix (ECM) accumulation, filtration membrane damage, and renal interstitial fibrosis (59). Transforming growth factor-*β*1 (TGF-*β*1) promotes the synthesis of ECM, prevents its degradation, and accumulates ECM by promoting adhesion between cells and matrix (60). Fibronectin (FN) is one of the key components of ECM, which can be applied to assess the extent of ECM accumulation. Ma et al. (33) recruited 464 patients and found that the miR-192 level in the DN group (especially in the albuminuria group) was significantly lower than the healthy controls (*P*< 0.05). The expression of miR-192 was negatively correlated with TGF-*β*1, FN, and urine albumin creatinine ratio (UACR). The authors concluded that there was a positive association between the low level of miR-192 and nephritic fibrosis in DN.

Histopathological examination is the gold standard for identifying multiple diseases. Krupa et al. reported that a low level of miR-192 was associated with the promotion of kidney fibrosis in DN (42). However, this finding was derived from kidney tissue samples, which was hard to implement for clinical practice. Mounting evidence shows that non-invasive sampling methods (i.e., serum and urine examination) are promising for the early detection of various diseases, including DN. Yang et al. (34) investigated 283 DN patients and found that the miR-192 level in serum significantly decreased in patients with DN, whereas in urine it increased with the progression of DN. Both of the differences were statistically significant. Therefore, the authors suggested that both serum and urinary miR-192 could be a potential biomarker of DN.

Though microalbuminuria was found to be an early sign of DN, some researchers wonder about its ability to precisely detect the disease progression of DN. So, it is necessary to find a diagnostic and predictive marker for DN. Given that circulating miR-192 might represent a potential novel source of non-invasive biomarkers for DN, more and more were designed for validating this finding. KAFAJI et al. (35) assessed 85 DN patients in Manama and found that lower miR-192 expression was observed in DN patients compared with healthy controls (*P*< 0.05). As a result, blood-based miR-192 was considered to serve as an effective biomarker for the early detection of DN. In line with this finding, Tayel et al. (36) evaluated the miR-192 expression in 229 patients and found that significantly lower levels of miR-192 were observed in patients with DN (particularly in the macroalbuminuria group) than in the controls (*P*< 0.05). In addition, marked downregulation of miR-126 was also detected in DN patients compared to in the control group (*P*< 0.05). Although effective, miR-192 had higher sensitivity (91%), specificity (94%), and area under the curve (0.967) values than that of miR-126. Tayel et al. (36) pointed out the potential role of miR-192 and miR-126 in the progression of DN and their prognostic value in the prevention of worsened progression to end-stage renal disease (ESRD). Consistently, a recent study conducted by Ren et al. (40) also reported the downregulation of miR-192 in patients with DN. Simultaneously, the expression of TGF-β1 was found to be up-regulated in DN patients. miR-192 interacted with some other miRNAs, their target genes mainly revolve around PTEN, PI3K/Akt, and MAPK signaling pathways. This transcriptional regulation mediated by miR-192 and TGF-β1 might participate in the fibrosis process of DN.

Since multiple microRNAs are associated with the development and progression of DN, Akpınar et al. (37) investigated the expression of ten microRNAs (miR-192-5p, miR-126-3p, miR-129-1-3p, miR-21-3p, miR-137, miR-29a-3p, miR-29b-3p, miR-29c-3p, miR-212-3p, and miR-320c) in 150 participants. The authors demonstrated that the expression of miR-192-5p in serum was significantly lower in the DN group than in the controls (*P*=0.027). Besides, the area under curve value was 0.717 for miR-192-5p for distinguishing the DN group from the control group, suggesting that this miRNA might involve in the development of DN.

Inconsistent with the above findings, Gong et al. (39) reported the opposite results which showed that the miR-192 level was increased in the serum samples of the DN patients (n=79). Different from the above included studies, the study population in Gong et al. was pediatric patients. The authors revealed that miR-192 expression negatively correlated with Alpha-Klotho (KL) levels in pediatric patients with prolonged duration of diabetes. KL was found to decrease by -246.8 pg/ml per each 1-unit increase in miR-192 relative ratio. KL, a known anti-aging protein, expresses in multiple tissues, with the highest expression in the kidneys (61). miR-192 might be an upstream regulator of KL (direct target gene) due to overexpression of miR-192 could significantly inhibit the KL expression in HK2 cells. The subsequent result showed that miR-192 targeted KL through its 3’UTR. Both miR-192 and KL together contributed to oxidative stress, inflammation, and senescence in DN development. In addition, they found that miR -192 expressions were stable among patients with shorter diabetes duration. In those with 12 years of diabetes, miR-192 levels elevated with time. Unlike the results suggesting miR-192 was significantly increased in adult DN patients or decreased in pediatric DN patients, Chien et al. demonstrated that no significant difference in serum was found regarding miR-192 expression in patients with or without DN. However, the authors found that serological miR-192 differed between microalbuminuria and overt proteinuria groups (P=0.0138), indicating that miR-192 might predict late DN progression.

### miR-192 expression in the urine of patients with DN

Among the ten included clinical studies, three of them investigated the expression of miR-192 in the urine of DN patients. Multiple miRNAs are found to enter the body fluid and blood-stream circulation (62). Thus, the detection of miRNAs in serum or body fluids (i.e., urine) plays an important role in the early diagnosis of various diseases, including DN (63). Similar to the findings of miR-192 expression in serum, its expression level in urine was also controversial among different studies. Two clinical studies showed that miR-192 expression was higher in the serum of the DN patients than in the controls, while a decreased level was found in the other study. Jia et al. (32) developed a small sample size with 80 participants and observed that the urine extracellular vesicle level of miR-192 was significantly elevated in patients with DN, especially in those with albuminuria. They also found that TGF-*β*1 levels were significantly correlated with miR-192 expression (*r* = 0.356, *P* = 0.005). Thus, both urinary extracellular vesicles miR-192 and TGF-*β*1 might serve as promising biomarkers of the early stage of DN. Consistent with Jia et al.’s findings, Yang et al. (34) reported that the miR-192 level in urine was increased, whereas in serum it decreased with the progression of DN (n=283). They further indicated that a combination with high levels of urine miR-192 and low levels of serum miR-192 had a higher specificity and lower misdiagnosis rate. However, Smith et al. (38) observed an opposite result that low miR-192 expression might be associated with the progression of DN. The authors detected that miR-192 expression fell from a 1.54-fold change in the control cohort in DN patients. MiR-192 was found to be specifically expressed in renal cortical tissue, and stored and excreted in urine as microvesicles (64). Therefore, kidney injury caused by DN might induce more microvesicles containing miR-192 through the urine. Nevertheless, since it is still being debated on the expression level of urinary miR-192 in DN patients, its diagnostic and predictive effects on DN still require further exploration.

### Molecular mechanisms of miR-192 IN DN

The aforementioned clinical studies suggested a causal association between miR-192 expression and DN, exploring the biological function of miR-192 and its potential mechanisms in DN might be profound for the researchers. MiR-192 was down-regulated in the serum of DN patients, which was reported in the majority of the clinical studies (6/8, 75%). However, the vast majority of experimental studies (14/18, 78%) demonstrated that miR-192 was up-regulated in a cell or animal model of DN (high glucose). Therefore, whether miR-192 played a protective or pathogenic role in the development of DN was still controversial among different studies. Since miRNAs function by interacting with their target genes, we summarized the 18 included *in vitro* and *in vivo* studies employing the different miR-192-targeted genes.

### Roles of ZEB1 and ZEB2 underlie the effect of miR-192 in DN genesis

An essential role for EMT exists in renal interstitial fibrosis in DN (65). The formation and deposition of EMT and ECM in renal tubular epithelial cells attribute to the pathogenesis of DN. The physiological function of miR-192 was found to be closely correlated to EMT. It is known that Zinc finger E-box-binding homeobox (ZEB) plays a key role in EMT, which is closely related to many aspects of life (66). ZEB family mainly comprises two proteins ZEB1 and ZEB2. Two zinc finger clusters in ZEB1 bind to DNA sequences specific to its function. There are mainly binding sites in the zinc finger region of the N-terminal zinc finger or the zinc finger region of the C-terminal zinc finger. In the downstream region of the zinc finger of ZEB1, phosphorylated receptor-activated SMAD is bound, so ZEB1 could regulate the signaling pathway of TGFβ (67). ZEB1 interacts with various miRNAs (i.e., miR-200c and miR-205), and together mediate the corresponding signaling pathways, such as TGFβ, hippo pathway, and wingless/integrated (Wnt). It is known that EMT plays a key role in the development of fibrosis, while ZEB1 is an important transcription factor of EMT. During the process of EMT, ZEB1 effectively promotes cell proliferation, migration, collagen synthesis, and fibrosis formation (68).

Five included studies demonstrated that ZEB1/ZEB2 might be a direct target of miR-192. Deshpande et al. (45) conducted an experimental study in cells and animals and subsequently validated it in human kidney tissues. They found an increased expression level of miR-192 and p53 and a decreased level of ZEB2 (the direct target for miR-192). TGF-b could promote the transcriptional activation of p53 through miR-192. This study showed that the TGF-b1–induced feedback amplification circuit between p53 and miR-192/ZEB2 contributed greatly to the pathogenesis of DN. Similar to this finding, Chen et al. (53) observed that miR-192 was increased in glomerular mesangial cells cultured with high glucose. High glucose levels could regulate both ZEB1 and monocyte chemotactic protein-1 (MCP-1) expression by upregulating the level of miR-192. Though lacking a luciferase reporter gene examination, the expression of miR-192 significantly increased after the targeting silencing of ZEB1, indicating ZEB1 might be the target of miR-192. miR-192/ZEB1 was considered to involve in the occurrence of the inflammatory reaction in DN development. Consistently, Putta et al. (44) also detected a high level of miR-192 in the mice and cell models of DN. Zeb1/2 (miR-192 targeted genes) were decreased by means of the TGF-β signaling. They further found that locked nucleic acid (LNA)–modified inhibitor of miR-192 dramatically increased Zeb1/2 expression and reduced the level of collagen, TGF-b, and fibronectin, therefore alleviating DN. Consistently, Yu et al. (54) reported that miR-192 elevated in a mouse model of DN. They next found that metformin or alcohol extract of Coreopsis tinctoria Nutt (AC) significantly decreased the expression of miR-192, which could alleviate the degree of renal fibrosis. The protective effect of AC on DN might be associated with the reduction of the expression level of miR-192 and upregulation of miR-192-targeted gene ZEB2. This protective effect may be attributed to the indirect modulation of the activity of the PTEN/PI3K/AKT pathway. The above four studies confirmed that miR-192 was up-regulated under high glucose conditions. Contrary to the above three included studies, Krupa et al. (42) reported that miR-192 was low in both patient tissue and HK-2 cells of the DN model. The author observed that upregulated ZEB1/ZEB2 (potential targeted genes for miR-192) and TGF-β and decreased levels of E-cadherin in the pathogenesis of DN. The downregulated miR-192 level could increase fibrosis and decline GFR in DN, which might induce by enhancing TGF-β–mediated downregulation of E-cadherin in proximal tubular cells. The above evidence indicated that ZEB1/ZEB2-associated EMT might contribute to the development of miR-21-mediated DN and the progression of miR-21-mediated DN.

### Roles of SIP1 underlie the effect of miR-192 in DN genesis

Smad-interacting protein 1 (SIP1), one of the two-handed zinc-finger proteins and the transcriptional regulators for E-cadherin expression, can interact with activated SMAD transcriptional cofactors (69, 70). Smads are the primary mediators of TGFβ signaling, which modulates the activity of SIP1 as a transcriptional repressor. In the present review, three experimental studies identified SIP1 might be the direct target for miR-192. All three studies demonstrated that miR-192 was up-regulated in numerous *in-vivo* and *in vitro* DN models. A previous study developed by Kato et al. (41) showed that SIP1 and δEF1 were down-regulated and Col1a2 was increased in DN mouse and mesangial cells with high glucose. In this study, the authors found that TGF-β led to the down-regulation of SIP1 (via miR-192) and δEF1 could cooperate to reinforce Col1a2 expression *via* derepression at E-box elements. δEF1 is an important inhibitor of E-cadherin. The cross-talk between E-box repressors (δEF1 and SIP1) contributes to the TGF-β-mediated collagen regulation in the pathogenesis of DN. Mao et al. (52) reported that astragaloside IV decreased miR−192, TGF-β1, Smad3, α-SMA, and collagen type 1, and elevated Smad7 in rats and RMCs cells model of DN. The therapeutic effect of Astragaloside IV on DN might be associated with the repression of excessive mesangial proliferation and renal fibrosis *via* the TGF-β1/Smad/miR-192 pathway. A more recent study (57) showed that TGF-β and miR-192 expression significantly elevated and SIP1 decreased in the DN group. Interestingly, the administration of troxerutin and insulin significantly reversed this tendency, indicating the renal-protective effects derived from these agents might be attributed to the inhibition of miR-192 expression and the elevation of SIP1 expression.

### Other targeted genes of miR-192 in DN genesis

Glucagon-like peptide-1 receptor (GLP1R) exists in various organs, including the liver, brain, pancreas, gut, and hypothalamus (71). In addition, GLP1R was also found to be highly expressed in the kidney, especially in renal tubular epithelial cells (72). Mounting evidence demonstrates that GLP1R is a pivotal pharmacological target for T2DM (73). Thus, GLP1R was believed to regulate both non-diabetic and diabetic renal fibrosis (74). Jia et al. (49) conducted an *in-vitro* study on HK-2 cells in the high glucose condition. The authors indicated the level of miR-192 was down-regulated and p53 expression was up-regulated. They found that exendin-4 downregulated cellular and secreted miR-192, therefore increasing the expression of GLP1R in a p53-dependent manner, which might ameliorate renal fibrosis. A subsequent *in-vitro* (HK-2) and *in-vivo* (rats) study (55) also suggested that miR-192-5p was elevated and its targeted gene GLP-1R was decreased in high-glucose-incubated human renal tubular epithelial cells and rat renal fibroblasts. The researchers next found that Icariin and dihydrotestosterone improved diabetic renal tubulointerstitial fibrosis by restoring autophagy *via* the miR-192-5p/GLP-1R pathway.

Early growth response factor 1 (Egr1), a transcription factor binding to DNA, has been found to abnormally express in diabetic mice (75). Besides, Egr1 is associated with the development of renal fibrosis (76). Multiple mechanisms are involved in the actions of Egr1 in diabetes mellitus-associated renal fibrosis, including elevating the expression of TGF-β, promoting the proliferation of mesangial cells, and accelerating the EMT process of renal tubular epithelial cells (77, 78). Thus, targeting with Egr1 might have promising effects for treating DN. Liu et al. (50) reported that miR-192 was down-regulated in both HK-2 cell and rat models of DN. Subsequently, the authors observed a tendency of high levels of Egr1 and low levels of TGF-β1 and FN in both cell and animal models. The biological effects exerted by miR-192 that could inhibit the progression of DN and protect DN rats from renal interstitial fibrosis might be mediated by decreasing TGF-β1 and FN. This study indicated that miR-192 could serve as an innovative and prospective therapeutic target for DN.

## Perspectives

In this review, conflicting results were identified among the included studies about the expression of miR-192 among serum, urine, renal tissues samples. Based on the 28 eligible studies, the level of miR-192 tend towards decrease in serum but increase in urine and renal tissues under the disease condition of diabetic nephropathy. One of the probable explanations was that miR-192 might release from other tissue types thus changing the actual level. Numerous studies (79, 80) indicated that serum miR-192 was downregulated in patients or animal models with diabetes mellitus. Consistently, we also found that serum miR-192 was decreased in diabetic nephropathy. It is known that the blood travel almost solely through the renal system. Therefore, the small molecules contained in the blood, like miR-192, may accumulate in the kidneys. Therefore, there is constant kidney accumulation of miR-192, leading to the high level of miR-192 in renal tissues and subsequently excrete with the urine. As a result, the expression of miR-192 may elevate in both kidney and the urine. On the other hand, miR-192 was found to be abundant in the renal glomerular and tubulointerstitial fibrosis. We speculated that renal fibrosis might encapsulate the small molecule “miR-192”, leading to an accumulation of miR-192 in the renal tissues. As aforementioned, serum miR-192 was downregulated in patients or animal models with diabetes mellitus, while diabetic nephropathy was considered to the vascular complication of diabetes mellitus. Thus, low serum miR-192 may be the early manifestation of diabetic nephropathy. Gradually, however, an increase of miR-192 level in the renal tissues and urine was detected with increased loading of circulating miR-192. According to this assumption, higher expression of miR-192 in both renal tissues and urine may indicate the progression of diabetic nephropathy. According to the above hypotheses, low serum miR-192 might be helpful for early diagnosis of DN, while higher miR-192 level in renal tissues and urine may suggest the progression of DN.

The challenges with the current knowledge about miR-192 in DN may be the inconsistent results of the expression of miR-192 in serum, urine, and renal tissues samples among the different included studies. However, these conflicting results might cause by the different stages of DN. Low serum miR-192 could be applied for the early prediction of DN (the early stage of DN), while the high miR-192 level in renal tissues and urine may imply the progression of DN (the late stage of DN). This hypothesis should be investigated in near future, especially for the well-designed clinical trials.

## Conclusion

To the best of our knowledge, this is the first review to summarize all the current evidence on the topic of the roles of miR-192 in DN. According to the 28 included studies, most of the clinical trials indicated miR-192 might be a protective factor for DN development and progression, while the majority of experimental studies suggested miR-192 might be a pathogenic factor for DN. Mechanistically, miR-192 interacts with various direct targeted proteins (i.e., ZEB1, ZEB2, SIP1, GLP1R, and Egr1) and signaling cascades, together contributing to the pathogenesis of DN. Since the results are conflicting and controversial between clinical trials and experimental studies, further investigations are still warranted to illustrate this inconsistent phenomenon. A clear understanding of the clinicopathological features and the molecular mechanisms may provide new insights into the therapeutic applications of miR-192 in predicting and treating DN. This review highlights the dual role of miR-192 in the pathogenesis of DN, which still needs more studies to verify its role in serving as a biomarker.

## Author contributions

XW, JL, and JC contributed to conceive and design the study. SZ performed the systematic searching. JL and HL extracted the data. XW and JC wrote the manuscript. XW and CC supervised the manuscript. All authors contributed to the article and approved the submitted version.
